# Immunotherapies in neuromyelitis optica: Bayesian network meta-analysis

**DOI:** 10.1007/s00415-025-13279-7

**Published:** 2025-08-08

**Authors:** Nevin John, Andy Lim, Shevita Ram Sunthar, Leon Zhang, Joan Cheng, Julie Boktor, Victor Chong, Henry Ma, Thanh G. Phan

**Affiliations:** 1 Department of Medicine, School of Clinical Sciences, Monash University, 246 Clayton Road, Clayton, VIC Australia; 2 Department of Neurology, Monash Health, Melbourne, VIC Australia

**Keywords:** Neuromyelitis optica spectrum disorder, Demyelinating disorders

## Abstract

**Background:**

There are numerous immunotherapies that are effective in preventing relapses in neuromyelitis optica spectrum disorder (NMO-SD). With head-to-head clinical trials between immunotherapies lacking, Bayesian network meta-analysis can be used to compare treatment interventions. Previous network meta-analyses have compared monoclonal antibodies but either not included newer complement inhibitors or earlier immunotherapies such as rituximab or tocilizumab.

**Objective:**

To compare immunosuppressive treatments used in relapse prevention in NMO-SD.

**Methods:**

PubMed, EMBASE and Scopus were searched for randomised controlled trials until 20th September, 2024. Search terms strategy included *neuromyelitis optica, antibody* and *relapse*. Randomised controlled trials testing immunotherapies used in relapse reduction in NMO-SD were included. Of 550 studies screened, 8 clinical trials initially met inclusion criteria. The study was performed according to PRISMA guidelines by multiple observers. Bayesian fixed-effect network meta-analysis was conducted. The primary outcome was time to relapse. The secondary outcome was annualised relapse rate. Sensitivity analysis was undertaken in seropositive patients. Treatments were ranked using a probability measure called surface under the cumulative rank curve (SUCRA).

**Results:**

Eight studies were included that contained a total 851 patients [716 (84%) seropositive]. There were six treatment interventions—ravulizumab, eculizumab, tocilizumab, rituximab, inebilizumab, satralizumab and the control arm (placebo/azathioprine). Ravulizumab was the ideal treatment (HR 0.00 (95%CrI 0.00–0.03), SUCRA 0.99) with a 98% probability of being the superior treatment in increasing time to relapse in NMO-SD. This was supported by secondary analysis of annualised relapse rate and the sensitivity analysis in seropositive patients.

**Discussion:**

These findings suggest that ravulizumab had the highest probability of being the most superior treatment in decreasing relapse risk in NMO-SD.

**Supplementary Information:**

The online version contains supplementary material available at 10.1007/s00415-025-13279-7.

## Introduction

Neuromyelitis optica spectrum disorder (NMO-SD) is a rare relapsing autoimmune disorder affecting the central nervous system encompassed by complement mediated astrocytopathy and inflammatory demyelination directed towards aquaporin water channels [1]. NMO-SD can be seropositive (80%) with the presence of pathogenic antibodies against aquaporin-4 water channels; or seronegative (20%) [1]. Eighty to ninety percent have relapsing disease characterised by the clinical attacks causing several typical clinical syndromes—optic neuritis, brainstem and area postrema syndromes, hemispheric involvement, narcolepsy and longitudinally extensive transverse myelitis with disability directly related to relapses [1, 2].

The long-term prevention of relapses is achieved through immune suppression. Previously, agents such as azathioprine, mycophenolate mofetil and rituximab, were used but more recently there have been several positive randomised controlled trials using monoclonal antibodies with differing mechanisms demonstrating significant efficacy in decreasing relapse risk. These include B cell depleting agents (inebilizumab, rituximab) [3–5], interleukin-6 drugs (satralizumab, tocilizumab) [6–8]; and complement inhibitors (ravulizumab, eculizumab) [9, 10]. These immunosuppressive agents reduce relapse risk by 60–90% compared to placebo (or active comparator azathioprine) [1]. However, it is not clear whether one treatment is superior to another as there no head to head randomised controlled trials of monoclonal antibodies; and no ranking of immunosuppressive treatments is available. Cost and feasibility issues mean that head-to-head or multi-arm trials clinical trials are unlikely to be completed. Bayesian network meta-analysis (NMA) can be used to investigate the comparative effectiveness of multiple treatments that have not been compared directly using a network-based approach. There have been several recent network meta-analyses in NMO-SD but these have compared recently approved mono-clonal antibodies and did not include older monoclonal antibodies (rituximab, tociliuzmab) [11], or did not include the most recently approved immunotherapy ravulizumab and used a frequentist approach [12–14]. We use Bayesian network meta-analysis to compare all available immunosuppressive treatments for NMO-SD.

## Methods

### Eligibility criteria

This meta-analysis conformed to the Preferred Reporting Items for Systematic Reviews and Meta-Analyses (PRISMA) Extension Statement for Reporting of Systematic Reviews Incorporating Network Meta-Analyses of Health Care Interventions [15]. We included clinical trials matching the following criteria: (1) participants: adult patients (≥ 18 years) diagnosed with NMOSD according to the Neuromyelitis Optica Diagnostic criteria [2], (2) intervention: the monoclonal antibodies ravulizumab, rituximab, eculizumab, inebilizumab, satralizumab, and tocilizumab, the immunosuppressants AZA, MMF, cyclophosphamide, tacrolimus. The comparator was placebo or an active comparator (3) outcomes: time to relapse, annualized rate of relapse (ARR), defined as the number of relapses divided by the time in years, the number of patients who experienced relapse (4) study type: Randomised controlled trials. We excluded studies matching at least one of the following criteria: (1) study type: conference abstracts, case reports, review articles, and noncomparative studies, retrospective or prospective studies; (2) studies with incomplete or unreported data; (3) studies not written in English; (4) studies specifically considering patients with the myelin oligodendrocyte glycoprotein autoimmune disease (MOGAD).

### Information sources

Two study authors (SR, LZ) independently searched MEDLINE, EMBASE, SCOPUS, clinicaltrials.gov and reference lists of relevant papers for clinical trials published up to September 20, 2024. Search terms included *neuromyelitis optica*, *antibody, relapse*. Only full published papers in English were considered. Reference lists of relevant articles and from experts in the field were involved in identifying relevant studies. Studies were managed through Covidence systematic review software [16]. Articles were included by consensus of the authors with any disagreements resolved by a third independent adjudicator.

Data included trial name, author, cohort start and end years, publication year, baseline EDSS, cohort size, seropositivity, number of events, number of events in seropositive groups, hazard ratios. The geometry of the network was described by a network diagram. Assessment of risk of bias was performed using the Cochrane Risk of Bias tool for randomized trials and completed independently by three study authors (JC, JB, VC) [17].

### Effect measures

Time to first relapse was the primary outcome measure. Secondary outcome measures were annualised relapse rate (ARR) which was the other outcome measure common to randomised clinical trials in NMO-SD. Relative treatments were expressed as hazard ratios with 95% confidence intervals. For ARR, the NMA was performed on the basis of the number of events over the total follow-up time with treatment exposure using a binomial regression model. Relative treatment effects were expressed as rate ratios (RRs) and 95% credibility intervals. A sub-group analysis was undertaken in those with seropositive NMO. As an additional analysis requested by reviewers, we compared treatments based on infection rates. We recorded number of patients with infections in each treatment group. This was also recorded for serious infections. Treatment rankings were estimated with surface under the cumulative ranking curve (SUCRA) value, which is the probability that a treatment has of being the best option [18]. The SUCRA is a summary measure of how likely each treatment is to be superior when compared with all other treatments in a network. Each treatment is given a probability of being ranked at each position, then the area under the curve of these cumulative probabilities is calculated as the SUCRA to give a summary measure of the full ranking distribution.

### Statistical analysis

We performed Bayesian NMA using R statistical software version 4.3.1 with the BUGSnet (Bayesian Inference Using Gibbs Sampling to Conduct an NMA) and JAGS (Just Another Gibbs Sampler) packages (R Project for Statistical Computing) [19]. Bayesian network meta-analysis is a method that allows comparison of multiple treatments, even if these treatments have not been directly compared with each other, by connecting head-to-head trials together into a network and making direct and indirect comparisons. From this, both probability distributions for each pair of comparisons and treatment rankings can be calculated. The BUGSnet library is recommended for NMA because it provides the necessary output required by various societies including the International Society of Pharmacoeconomics and Outcomes–Academy of Managed Care Pharmacy–National Pharmaceutical Council [19]. NMA was used because of the ability to rank treatments using the SUCRA value [18]. Assessment of inconsistency, which refers to the agreement between indirect and direct evidence within a network, was tested using the inconsistency model [20], as recommended by the National Institute for Health and Clinical Excellence Decision Support Unit [21], Statistical significance was considered a two-sided *P* < 0.05.

## Results

### Study characteristics

**Fig. 1:**
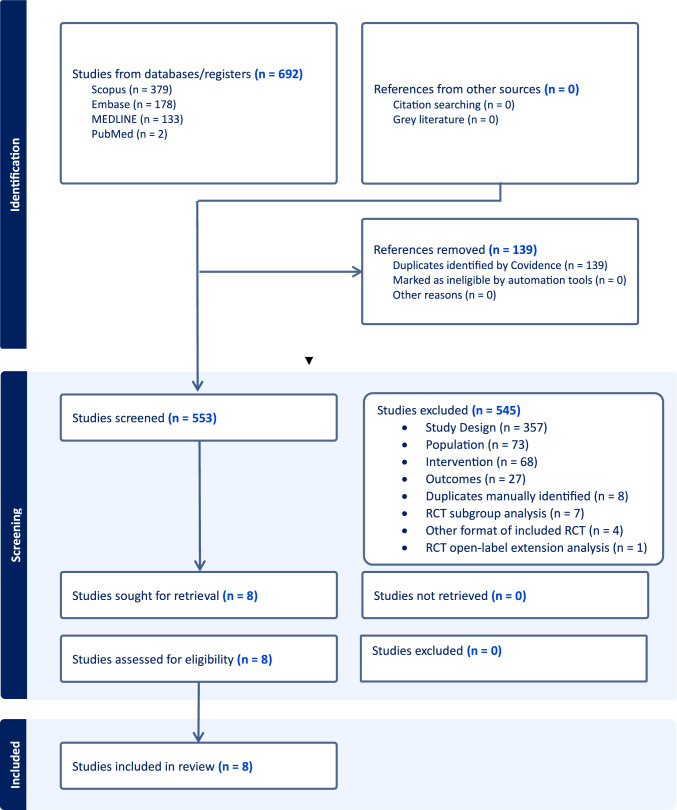
Preferred reporting items for systematic reviews and meta-analyses flow diagram

Eight clinical trials were identified that met inclusion criteria with the majority of studies excluded for not being randomised controlled trials (Fig. 1). The 8 clinical trials included 851 participants of which 716 (84%) were seropositive and 135 (16%) were seronegative. Five randomised controlled trials included both seropositive and seronegative patients, whilst 3 included only seropositive NMO-SD. Six randomised controlled trials used placebo as the comparator whilst 2 used an active comparator, azathioprine [3, 6]. All used the same outcome measure using a time to event analysis using the primary outcome measure of time to relapse except for the study of rituximab vs azathioprine by Nikoo et al. which used annualised relapse rate as the primary outcome measure. The study characteristics are shown in Table 1. The network diagram is shown in Fig. 2 in supplement 1.

**Table 1:** Study characteristics

Study	Author	Years of study	Intervention	Follow-up (months)	Number in group	Relapses (*n*)	Seropositive (%)	Relapses in seropositive (*n*)	Baseline EDSS (mean)	Age (sd)	Female (%)
N-MOMENTUM	Cree	2015–2018	Inebilizumab	6.5	174	21	161 (93)	18	3.8	43 (12)	159 (91)
			Placebo	6.5	56	22	52 (93)	22	4.2	43 (14)	50 (89)
PREVENT	Pittock	2014–2017	Eculizumab	48.7	96	3	96 (100)	3	4	44 (13)	88 (92)
			Placebo	48.7	47	20	47 (100)	20	3.9	45 (13)	42 (89)
TANGO	Zhang	2017–2018	Tocilizumab	13.8	59	8	50 (85)	6	4.7	48 (13)	55 (93)
			Azathioprine	13.8	59	28	53 (90)	25	4.8	45 (15)	53 (90)
SAKURASTAR	Traboulsee	2014–2017	Satralizumab	18.0	63	19	41 (65)	9	3.9	45 (12)	46 (73)
			Placebo	18.0	32	16	23 (72)	13	3.7	41 (11)	31 (97)
RIN-1	Tahara	2014–2017	Rituximab	16.6	19	0	19 (100)	0	3.4	51 (13)	17 (89)
			Placebo	16.6	19	7	19 (100)	7	4	50 (22)	19 (100)
SAKURA-SKY	Yamamura	2014–2018	Satralizumab	24.8	41	8	27 (66)	3	3.8	41 (16)	37 (90)
			Placebo	24.8	42	18	28 (67)	12	3.6	43 (12)	40 (95)
Azathioprine and rituximab in NMO	Nikoo	2015–2016	Azathioprine	12.0	46	1.02	25 (54)	N/A	2.4	32 (9)	38 (83)
			Rituximab	12.0	40	1.3	17 (43)	N/A	3.3	34 (9)	34 (85)
CHAMPION-NMOSD	Pittock	2019–2022	Ravulizumab	27.7	58	0	58 (100)	0	3.3	47 (14)	52 (90)

*EDSS* Expanded disability status scale

### Primary outcome: time to relapse

**Fig. 2:**
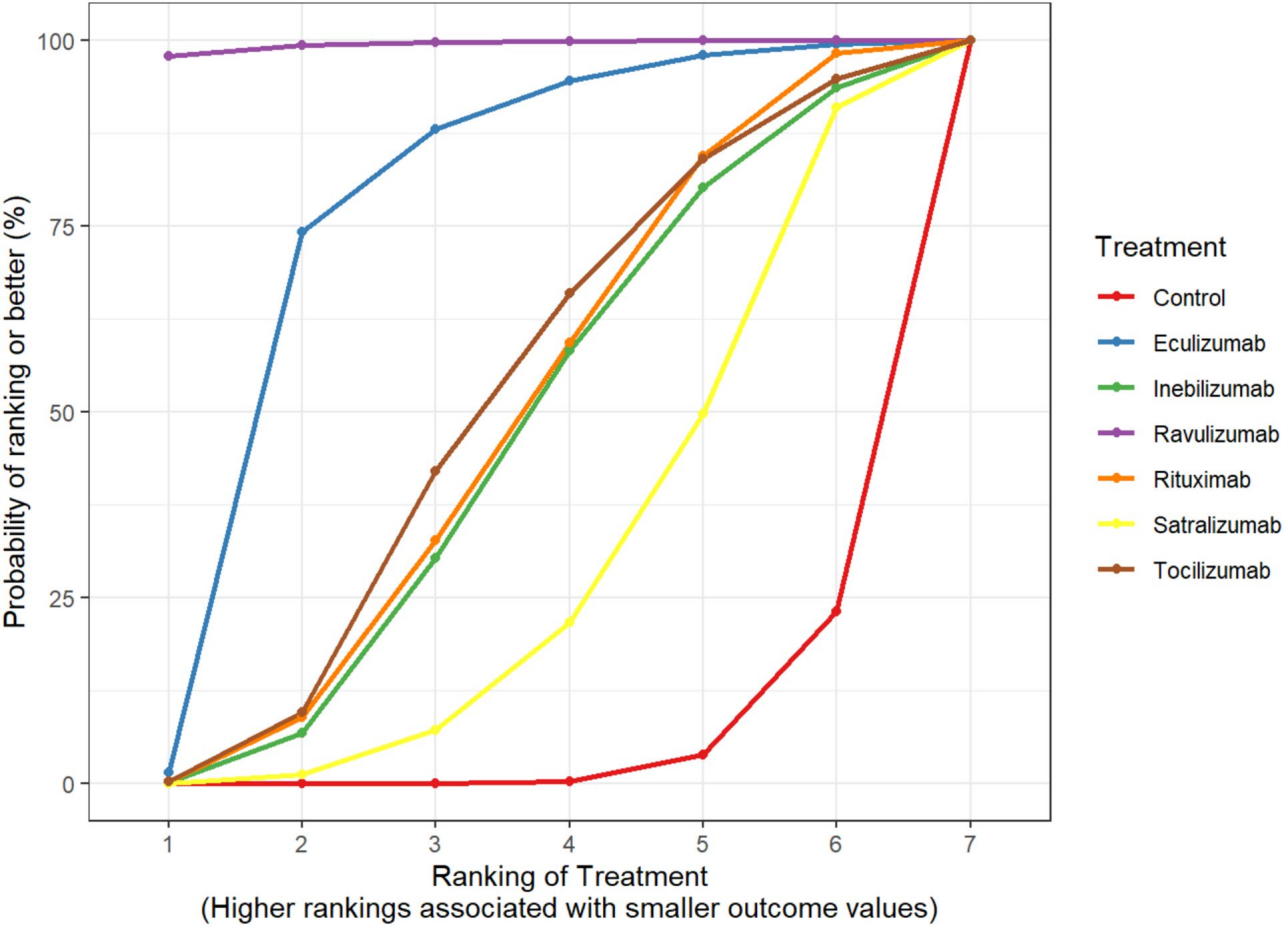
Treatment rankings for time to relapse

**Fig. 3:**
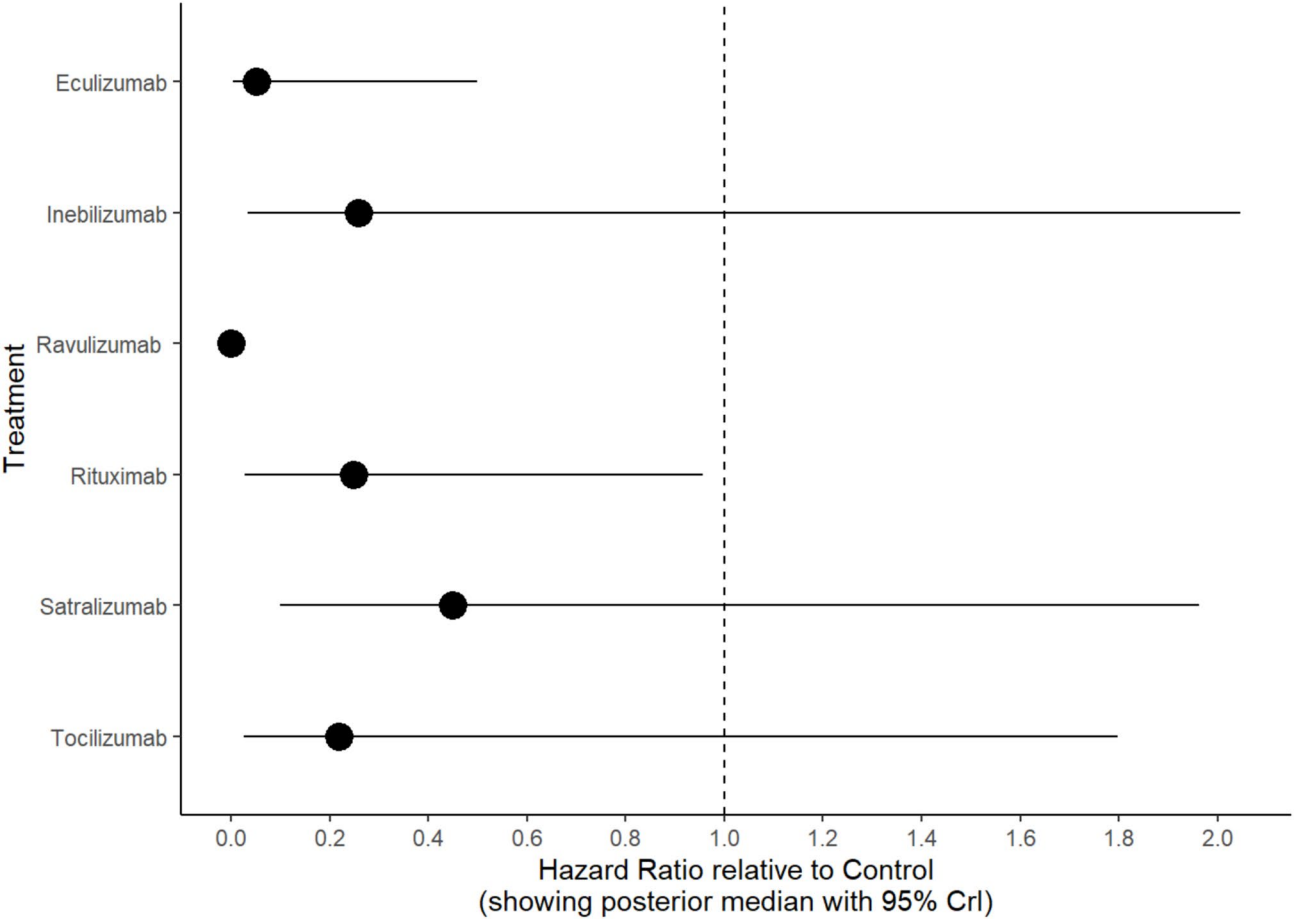
Forest plot of immunotherapies compared with traditional treatment

When ranking the treatments for the primary outcome of time to relapse, ravulizumab consistently ranked first as the ideal immunotherapy (HR 0.00 (95% CrI 0.00–0.03), SUCRA 0.99). The other complement inhibitor eculizumab was ranked second (HR 0.05 (95% CrI 0.00–0.50); SUCRA 0.76). The third ranked immunotherapy was tocilizumab (HR 0.22 (95% CrI 0.03–1.80), SUCRA 0.49) followed by the B cell depleting agents rituximab (HR 0.25 (95% CrI 0.03–0.96), SUCRA 0.47) and inebilizumab (HR 0.26 (95% CrI 0.03–2.05), SUCRA 0.45), respectively (Table 2, Fig. 2). The forest plot of the eight interventions compared to control is shown in Fig. 3. Compared to control, the HR were compatible with those previously published in the pivotal phase III randomised controlled trials with ravulizumab eculizumab and tocilizumab demonstrating the greatest reduction in time to relapse compared to control. When comparing treatments head-to-head, ravulizumab was superior to all other interventions (HR 0.0, varying 95% credibility intervals—see Table 2). No other significant results were seen in indirect comparisons between treatment interventions.

**Table 2:** Treatment rankings

	HR (95% CrI)	SUCRA
Time to relapse		
- Control		4.54
- Eculizumab	0.05 (0.00, 0.50)	75.92
- Inebilizumab	0.26 (0.03, 2.05)	44.90
- Ravulizumab	0.00 (0.00, 0.03)	99.46
- Rituximab	0.25 (0.03, 0.96)	47.31
- Satralizumab	0.45 (0.10, 1.96)	28.46
- Tocilizumab	0.22 (0.03, 1.80)	49.45
Annualized relapse rate		
- Control		10.39
- Eculizumab	0.06 (0.00, 2.36)	69.31
- Inebilizumab	0.31 (0.01, 7.35)	44.33
- Ravulizumab	0.00 (0.00, 0.05)	98.92
- Rituximab	0.27 (0.01, 1.81)	48.40
- Satralizumab	0.50 (0.05, 4.77)	31.82
- Tocilizumab	0.27 (0.01, 6.75)	46.81
Time to relapse—seropositive patients		
- Control		6.96
- Eculizumab	0.06 (0.00, 3.28)	56.87
- Inebilizumab	0.26 (0.01, 11.90)	35.26
- Ravulizumab	0.00 (0.00, 0.00)	92.08
- Rituximab	0.00 (0.00, 0.02)	90.34
- Satralizumab	0.31 (0.02, 4.44)	31.53
- Tocilizumab	0.24 (0.00, 10.79)	36.98

*HR* hazard ratio, *CrI* credibility interval, *SUCRA* surface under the cumulative ranking curve

### Secondary outcomes: annualised relapse rate

All clinical trials were included in the secondary analysis of annualised relapse rates. The results were the same as those of the primary analysis with ravulizumab being ranked as the ideal treatment in reducing relapse rate in NMO-SD (HR 0.00 (95% CrI 0.00–0.05), SUCRA 0.99) (Table 2, Fig. 5 in Supplement 1) and ravulizumab being superior in decreasing annualised relapse rates compared to all other interventions.

### Sub-group analysis: seropositive subgroup

The subgroup analysis of seropositive patients (*n* = 674) was derived from 7 trials as Nikoo et al. did not report the relapse rates in seropositive participants and was therefore excluded. Ravulizumab again had the highest probability of being the first ranked treatment (HR 0.00 (95% CrI 0.00–0.00), SUCRA 0.92) in increasing time to first relapse in NMO-SD. Rituximab was ranked the second therapy (HR 0.00 (95% CrI 0.00–0.02), SUCRA 0.90) followed by eculizumab (HR 0.06 (95% CrI 0.00–3.28), SUCRA 0.57). In comparisons with other treatments, ravulizumab and rituximab were superior to all other interventions (Table 2, Fig. 6 in Supplement 1).

3.5: Safety analysis on infection rates.

Our results did not identify any statistically significant differences in infection rates between the different immunotherapies (see Fig. 7 in supplement 1). We were unable to compare serious infection rates due to the sparsity of this data.

### Inconsistency, risk of bias

Testing for inconsistency did not reveal large differences between direct and indirect estimates. Individual trial bias demonstrated low risk for the majority of studies (Fig. 8 in Supplement 1). Studies by Nikoo et al. showed some concerns owing to lack of blinding [3]. The CHAMPION-NMOSD trial was an externally controlled study using the placebo group from the PREVENT study [9].

## Discussion

In a network meta-analysis of monoclonal antibodies used for NMO-SD, ravulizumab was consistently shown to be highest ranked suggesting that it is likely to be the optimal treatment for reducing relapse risk. We use a Bayesian approach to demonstrate for the first time, the superiority of ravulizumab and treatment rankings in an analysis that includes recently approved monoclonal antibodies but also older monoclonal antibodies—rituximab and tocilizumab.

Our analysis demonstrated that the two complement inhibitors ravulizumab and eculizumab had the highest probability of being the top two ranked therapies respectively. This suggests that complement inhibition may be superior to B cell depletion or IL-6 receptor blockage in relapse prevention. Complement activation, particularly via the classic pathway has been demonstrated to be a key and potentially early component of NMO-SD pathogenesis. The two complement inhibitors eculizumab & ravalizumab block the cleavage of C5 to C5a and C5b thereby preventing damage mediated by C5a (potent anaphylatoxin) and the terminal pathway leading to membrane attack complex formation [22]. Furthermore, the complement system can bridge the innate and adaptive immune systems by modulating activation of both B and T cells [23]. We postulate that the prevention of these multiple pathways that cover direct astrocyte destruction and the lesser role of reducing B/T cell stimulation may explain the superior ranking of the complement inhibitors.

It is important to note the CHAMPION-NMO-SD trial of ravulizumab did not use a placebo arm and used the placebo arm of the PREVENT trial [9]. As a result of this, in the analysis we incorporated ravulizumab as one arm of a multi-arm study with a common placebo arm. Our findings are in keeping with the only network meta-analysis by Clardy et al. that has incorporated ravulizumab which showed ravulizumab to be comparable to eculizumab and superior to inebilizumab and satralizumab in relapse prevention in NMO-SD [11].

Tocilizumab, an IL-6 receptor antagonist was the third ranked treatment whilst its IL-6 counterpart, satralizumab was the 6th ranked treatment. Tocilizumab was not studied in the most recent NMA of NMO-SD by Clardy et al. and previous NMA were published before ravulizumab was published [11–14]. Tocilizumab was studied in a randomised controlled trial against azathioprine (TANGO) of highly relapsing NMO-SD and contained 90% seropositive patients decreasing time to first relapse by 76% (HR 0.24) [6]. The inclusion criteria were very similar to the more recent clinical trials such as N-MOMENTUM, PREVENT with a pre-specified level of minimum baseline relapse activity prior to randomisation [5, 10]. An active comparator azathioprine was used here and those commencing azathioprine at randomisation were given concomitant immunosuppressive therapy until week 24 making any potential treatment effect of tociliuzmab potentially greater given the majority of other studies used a placebo comparator. Its IL-6 blocking counterpart, satralizumab was the 6th ranked therapy with two studies of similar inclusion criteria showing similar results but minimal effect in those with seronegative NMO. However, the proportion of seronegative participants in SAKURA-STAR and SAKURA-Sky were 65–72% across both studies compared to 90% in TANGO [6–8]. IL-6 clearly plays a significant role in NMO-SD as evidenced by increased IL-6 in CSF, serum and increases during times of disease activity; and positive clinical trials of anti-IL-6 receptor blockers but it is possible that IL-6 activity in seronegative NMO-SD may differ compared to seropositive disease.

The B-cell depleting agents, rituximab (anti CD-20) and inebilizumab (anti CD19) were the 4th and 5th ranked treatments respectively. Rituximab is an older immunotherapy and has been used off label in many countries for NMO-SD. It has been studied in two randomised controlled trials—against placebo (RIN-1) and azathioprine [3, 4]. Neither included participants on the basis of relapse activity prior to randomisation but in the RIN-1 study, 58–63% had a relapse in preceding 2 years with an ARR 1.4 in rituximab arm v 0.7 in placebo. The study using azathioprine as an active comparator used ARR as the primary endpoint and included a higher proportion of seronegative participants in each arm (42–54%) [4]. However despite these differences, the results between the two B-cell depleting agents was similar. This is also similar to a previous Bayesian NMA completed by Yin et al. who showed that rituximab was the first ranked choice by SUCRA in reducing ARR [13, 20]. However, this study included RCT, prospective and retrospective studies and the primary outcome analysis of ARR did not include inebilizumab [13]. Our results provide indirect evidence that B cell depletion using either rituximab or inebilizumab are also effective options in relapse prevention in NMO-SD.

In a post-hoc analysis of infection rates, we were unable to detect differences between treatment interventions. The previous study by Clardy et al. did not examine safety outcomes [11]. There are several limitations when interpreting our results. First, there were differences in the reporting of infection rates. For example: TANGO, CHAMPION, PREVENT all reported infections that occurred in > 10% participants, RIN-1 study reported infections occurring in > 20% patients, whilst the remainder did not set minimum frequency to report infections (except N-MOMENTUM study of inebilizumab where infections ≥ 2.5% were reported) [3–10]. Second, due to relative sparsity of data we were unable to analyse whether there were differences in serious infection rates between the different treatments and, therefore, no conclusions can be drawn about this.

### Strengths and limitations

The strengths of the study are the use of Bayesian network meta-analyses that enable indirect comparisons to enable treatment ranking that otherwise is not possible. We expand on previous literature and incorporate all available monoclonal antibodies that have previously not been present in previous NMA as they were published prior to the availability of ravulizumab or not included in the analysis (rituximab, tocilizumab) [11–14]. In particular, our incorporation of rituximab, which has widespread off-label usage, is important to understand its treatment ranking in the current landscape as newer monoclonal antibodies such as ravalizumab, eculizumab and satrilizumab are not yet approved for use in a number of countries. There are several limitations to the study—Bayesian network meta-analysis uses indirect comparisons and need to be interpreted as such and is not a substitute for randomised controlled trials. However, in the current era Bayesian NMA presents an approach for treatment comparison that would otherwise not occur due to feasibility and cost of repeated head-to-head randomised controlled trials. Another limitation of the study is smaller number of trials per comparison and the relatively small sample sizes in the individual arms of the respective trials. Satralizumab and rituximab each had two randomised control trials respectively; the rest of the treatment interventions had only 1 randomised clinical trial. However, there was overlap with the mechanisms of the treatment agents with treatments grouped into being B-cell depleting (rituximab, inebilizumab), anti-IL-6 (satralizumab, tocilizumab) and complement inhibition (eculizumab, ravalizumab). The highest sample size was for the inebilizumab treatment group with 174 with rest being below 100 and the lowest being 19 in the RIN-1 study of rituximab. However, each trial recruited to target with all except 1 having the requisite number of adjudicated events (relapses) to be adequately powered. The smaller sample sizes in the clinical trials are an inherent reflection of the relatively low prevalence/incidence of this disease and low sample sizes needed given the natural history of the disease and the severity of the relapses. The subgroup analysis of seropositive groups demonstrated a similar result with ravulizumab but rituximab was shown to be second ranked treatment. This was an effect of not including the Nikoo study which did not state the relapse rate in those that were seropositive and, therefore, favourably weighted rituximab. Furthermore, the only seropositive group that reported relapses did not experience a relapse and we are, therefore, unable to conclude whether rituximab is similar in efficacy to ravulizumab. Differences in relapse inclusion criteria are unlikely to be of significance—the studies that did not mandate minimum relapse activity prior to randomisation had baseline ARR that was similar to those that did incorporate relapse activity prior to randomisation. Last, combining placebo and azathioprine arms into a single ‘control’ group introduces heterogeneity, but allowing for separation of traditional treatments introduces added network complexity and artefact and was considered a reasonable compromise.

In summary, the two complement inhibitors, ravulizumab and eculizumab were shown to be the highest ranked treatments in relapse prevention in NMO-SD. Our findings inform treatment decision making from an efficacy perspective and need to be balanced against safety when deciding on an appropriate immunosuppressive strategy in NMO-SD.

## Supplementary Information

Below is the link to the electronic supplementary material.

Supplementary file1 (PDF 281 KB)

## Data Availability

These data are available from the publications related to each clinical trials. Data can be accessed by researchers whose proposed use of the data has been approved. The can be made available with investigator support.
