# Supplementary material for: Immunotherapies in neuromyelitis optica: Bayesian network meta-analysis
Source: J Neurol. 2025 Aug 8;272(9):563. doi: 10.1007/s00415-025-13279-7 (PMC12331795; doi:10.1007/s00415-025-13279-7)
Supplement: Supplementary file 1 — Supplementary file1 (PDF 281 KB) [file 415_2025_13279_MOESM1_ESM.pdf]

Online Supplement 1.

This supplement includes:

eFigure 2. Network diagram

eFigure 5. Treatment rankings for annualised relapse rate

eFigure 6. Treatment rankings for time to relapse in seropositive patients

eFigure 7. Forest plot of immunotherapies compared with traditional treatment for infection rates

eFigure 8. Risk of bias grading

eFigure 2. Network diagram

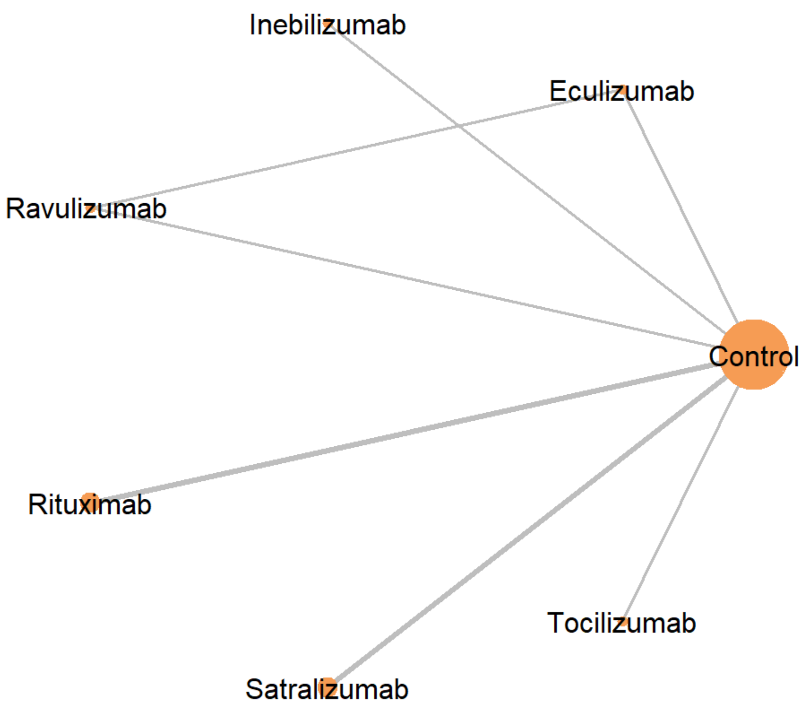

eFigure 5. Treatment rankings for annualised relapse rate

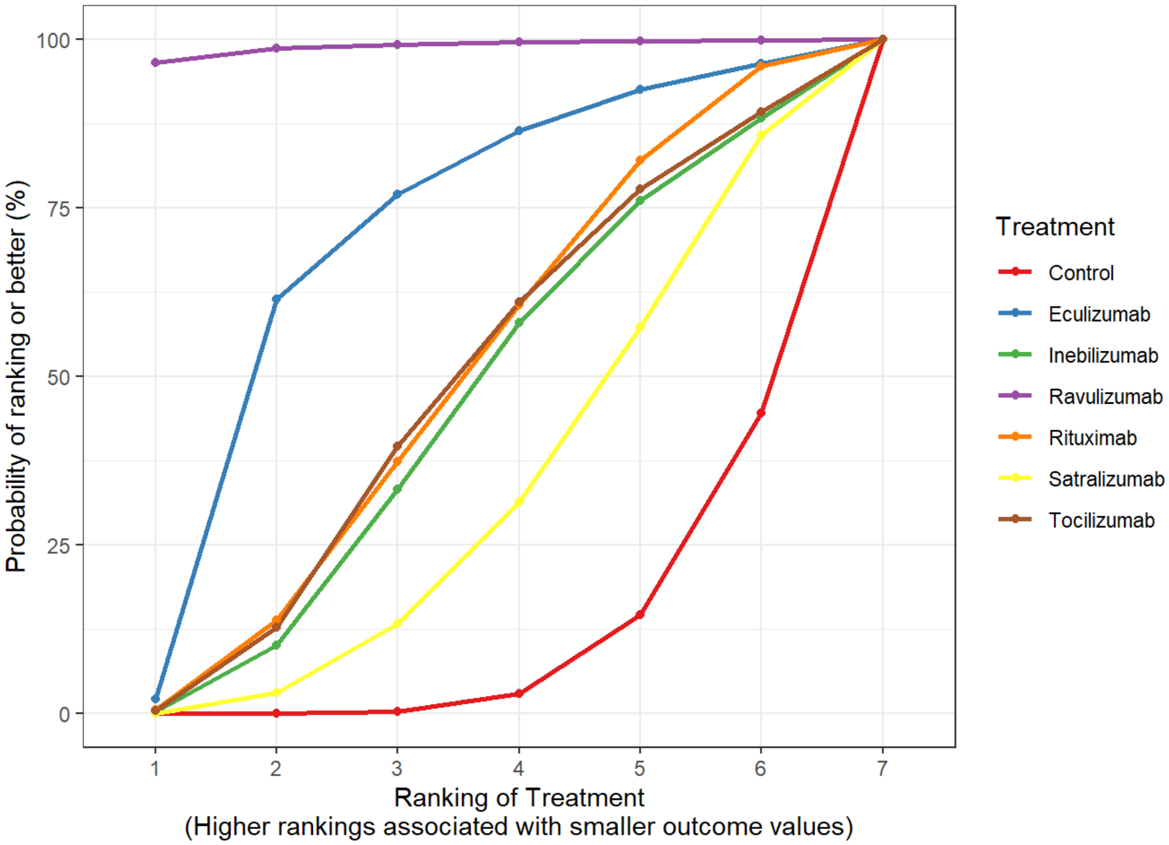

eFigure 6. Treatment rankings for time to relapse in seropositive patients

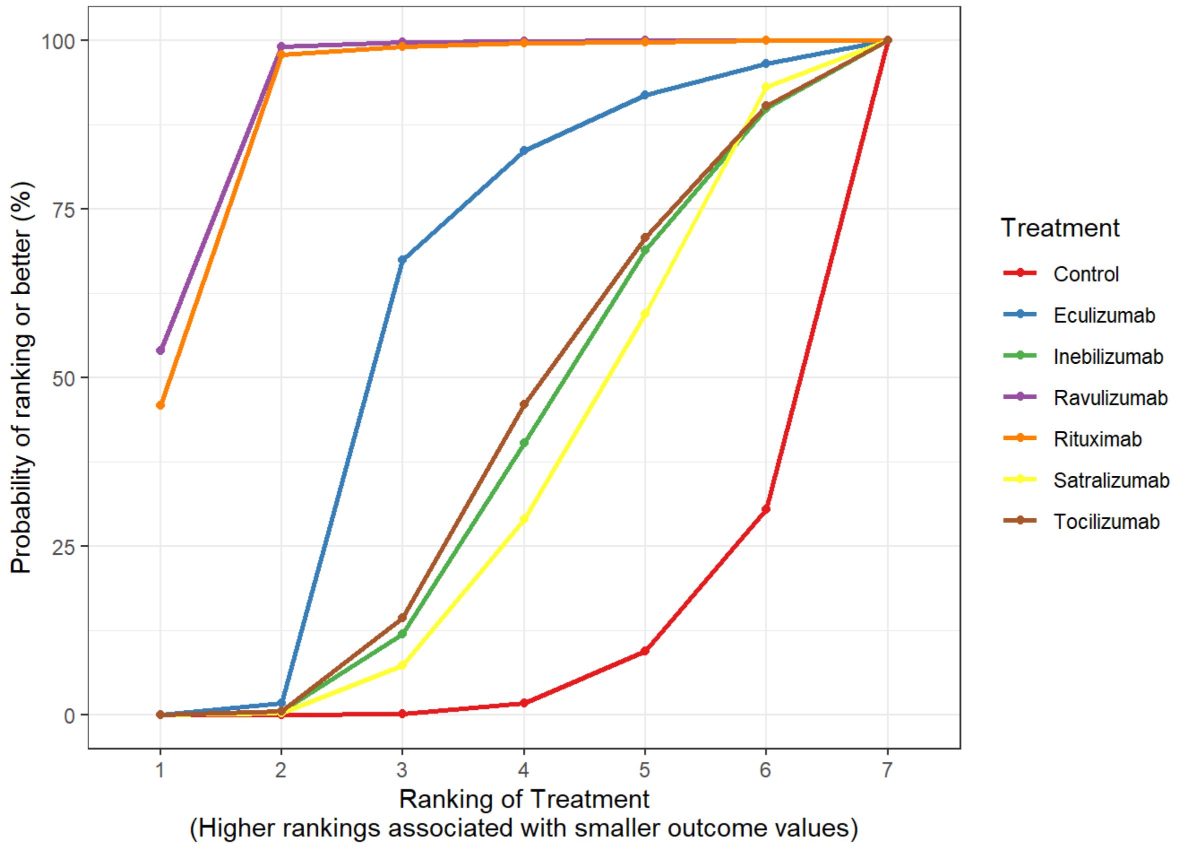

eFigure 7. Forest plot of immunotherapies compared with traditional treatment for infection rates

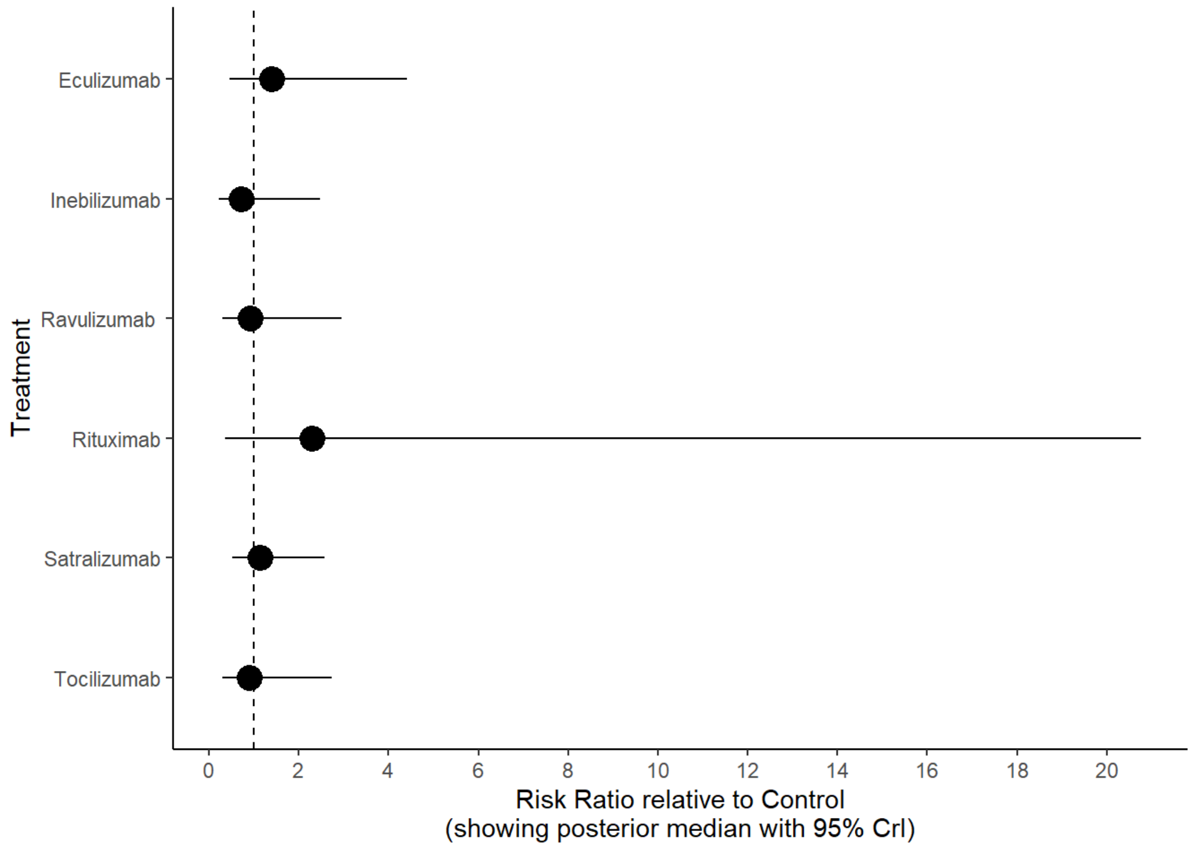

eFigure 8. Risk of bias grading

Study	Author	Years of study	D1	D2	D3	D4	D5	Overall
N-MOMENTUM	Cree	2015-2018	+	+	+	+	+	+
PREVENT	Pittock	2014-2017	+	+	+	+	+	+
TANGO	Zhang	2017-2018	+	+	+	+	+	+
SAKURASTAR	Traboulsee	2014-2017	+	+	+	+	+	+
RIN-1	Tahara	2014-2017	+	+	+	+	+	+
SAKURA-SKY	Yamamura	2014-2018	+	+	+	+	+	+
AZATHIOPRINE v RITUXIMAB	Nikoo	2015-2016	+	!	+	!	+	!
CHAMPION-NMOSD	Pittock	2019-2022	-	+	+	+	+	!

+	Low risk
!	Some concerns
-	High risk

D1	Randomisation process
D2	Deviations from the intended interventions
D3	Missing outcome data
D4	Measurement of the outcome
D5	Selection of the reported result
